# Novel Ensemble Feature Selection Approach and Application in Repertoire Sequencing Data

**DOI:** 10.3389/fgene.2022.821832

**Published:** 2022-04-26

**Authors:** Tao He, Jason Min Baik, Chiemi Kato, Hai Yang, Zenghua Fan, Jason Cham, Li Zhang

**Affiliations:** ^1^ Department of Mathematics, San Francisco State University, San Francisco, CA, United States; ^2^ Helen Diller Family Comprehensive Cancer Center, University of California, San Francisco, San Francisco, CA, United States; ^3^ Department of Medicine, University of California, San Francisco, San Francisco, CA, United States; ^4^ Department of Medicine, Scripps Green Hospital, La Jolla, CA, United States; ^5^ Department of Epidemiology and Biostatistics, University of California, San Francisco, San Francisco, CA, United States

**Keywords:** feature ensemble, VJ gene usage, repertoire sequencing data, high-dimensional data, COVID-19, adaptive immune system

## Abstract

The T and B cell repertoire make up the adaptive immune system and is mainly generated through somatic V(D)J gene recombination. Thus, the VJ gene usage may be a potential prognostic or predictive biomarker. However, analysis of the adaptive immune system is challenging due to the heterogeneity of the clonotypes that make up the repertoire. To address the heterogeneity of the T and B cell repertoire, we proposed a novel ensemble feature selection approach and customized statistical learning algorithm focusing on the VJ gene usage. We applied the proposed approach to T cell receptor sequences from recovered COVID-19 patients and healthy donors, as well as a group of lung cancer patients who received immunotherapy. Our approach identified distinct VJ genes used in the COVID-19 recovered patients comparing to the healthy donors and the VJ genes associated with the clinical response in the lung cancer patients. Simulation studies show that the ensemble feature selection approach outperformed other state-of-the-art feature selection methods based on both efficiency and accuracy. It consistently yielded higher stability and sensitivity with lower false discovery rates. When integrated with different classification methods, the ensemble feature selection approach had the best prediction accuracy. In conclusion, the proposed novel approach and the integration procedure is an effective feature selection technique to aid in correctly classifying different subtypes to better understand the signatures in the adaptive immune response associated with disease or the treatment in order to improve treatment strategies.

## Introduction

The adaptive immune system is responsible for recognizing and eliminating antigens originating from infection and disease. It recognizes antigens via an immense array of antigen-binding antibodies (B-cell receptors, BCRs) and T-cell receptors (TCRs), the immune repertoire. The interrogation of immune repertoires is highly relevant for understanding the adaptive immune response in autoimmunity, malignancy, and infection (Miho et al., 2018). Adaptive immune receptor repertoire sequencing (Rep-seq) has driven the quantitative and molecular-level profiling of immune repertoires, thereby revealing the high-dimensional complexity of the immune receptor sequence landscape. The advancement in high-throughput next-generation sequencing (NGS) technology has allowed researchers to sequence the immune repertoire profile from a single sample of blood or tissue.

Identification of prognostic and predictive features among high-throughput sequencing data is of high clinical relevance. Because individuals share almost no exact TCR/BCR nucleotide sequencing, TCR/BCR sequencing cannot be directly compared between different patient groups on the clonal level. However, TCR and BCR are the products of somatic V(D)J gene recombination, plus the addition/subtraction of nontemplated bases at recombination junctions. Thus, individuals share V(D)J genes, which allows for direct comparison of V and J gene usage across different patients. Therefore, it will enable researchers to directly obtain statistical inferences across subjects to provide insight into TCR/BCR repertoire with clinical characteristics and outcomes.

Though preliminary analysis using Random Forest reveal has been used to identify differentially expressed VJ genes in distinct disease types such as melanoma and prostate cancer (Cham et al., 2020), it is limited by the instability of feature selection due to the small sample size and sporadic gene usages. It has been shown that selecting the right set of features for classification and/or prediction can improve the performance of supervised and unsupervised learning, reduce computational costs such as training time or required resources, and mitigate the curse of dimensionality in the case of high-dimensional input data. Computing and using feature importance scores are also necessary steps towards model interpretability. In this paper, we introduce an ensemble feature selection strategy to select the significant V and J genes that can distinguish subjects in different groups defined by clinical characteristics, clinical treatment, or outcomes. Ensemble learning combines the results from multiple approaches, instead of simply using a single method, built on the rationale of “two heads are better than one.” However, it has been primarily used in the classical prediction task of machine learning and has successfully proven its effectiveness. For example, boosting (Schapire and Freund, 2013) and bagging (Breiman, 1996) (the Random Forest is a particular case of bagging) are two popular machine learning algorithms based on the ensemble idea, where aggregating multiple tree learners make the final prediction. Recently, it has become more and more popular to use pseudo-variables (e.g., permutation copies, knockoff copies) to assist variable selection, where artificial variables (independent of the response variable) will be generated (Candès et al., 2018). The advantage of introducing pseudo-variables is that they can help reduce the false-positive rate because they are designed to be inactive and provide additional information. Here, we considered implementing ensemble learning to improve the performance of feature selection based on pseudo-variables. Simulation studies were conducted to evaluate the efficiency and accuracy of the proposed procedure in addition to real data analysis by comparing our approach with current feature selection approaches.

## Materials and Methods

### European COVID-19 Data

The TCR-seq data used includes a cohort of patients who recovered after COVID-19 with mild to moderate disease courses (*n* = 19) and a cohort of age-matched healthy donor cohort (*n* = 39) that tested negative for COVID-19 antibodies. The clinical characteristics of the patients and sequencing information were shown in (Schultheiß et al., 2020) (gateway.ireceptor.org; Study ID: IR-Binder-000001). The median number of unique clonotypes was 9,431 (ranging from 589 to 35065) for healthy donors. Recovered patients had a median read depth of 72,152 ranging from 21,683 to 290,424. There was a total of 708 unique VJ gene combinations across both cohorts. VJ gene usage was defined as the number of clonotypes that utilize a particular combination of V and J genes normalized by the number of unique clones. Table 1 presents the summary statistics for the TCR sequences.

**TABLE 1:** Summary of TCR sequences in the real datasets.

European COVID-19 data [median (range)]	Recovered COVID patients (n = 19)		Healthy donors (n = 39)	
Number of unique clonotypes	17441 [6073,35065]		7952 [589,15271]	
Clonal counts	185758 [45066, 251020]		62429 [21683, 290424]	
VJ gene usage	1.1 × 10^−3^ [0, 0.816]		0.4 × 10^−3^ [0, 0.283]	
VJ gene usage for the selected 11 genes	0.010 [0, 0.816]		0.002 [0, 0.195]	
Lung Cancer Data	Longer survivors (n = 17)		Shorter survivors (n = 33)	
(median [range])	Baseline	Post-treatment	Baseline	Post-treatment
Number of unique clonotypes	6,144 [1,104,17876]	5,920 [403,13039]	4,708 [840, 13200]	3,737 [943,13839]
Clonal counts	206440 [1543567,3994587]	2028347 [1502019, 2718355]	2322713 [1483854,6956035]	2282314 [1348433, 7944974]
log_2_ (ratio of VJ gene usage)	0 [-16.31,15.60]		0 [-15.96, 16.00]	
log_2_ (ratio of VJ gene usage) of the selected 9 genes	0 [-13.14,12.88]		0 [-12.59,12.69]	

### Lung Cancer Data

The 686 TCR VJ gene combinations of 50 non-small cell lung cancer (NSCLC) patients receiving durvalumab enrolled in a Phase I trial (NCT01693562, 14 September 2012) were included for analysis. The median number of unique clonotypes was 4,994 (ranging from 403 to 17,876). In order to explore the treatment effect, here we considered using log_2_ transformed ratio of VJ gene usage after the treatment vs. the usage at baseline, where the VJ gene usage is defined as above. The clinical characteristics of the patients and sequencing information were shown in (Naidus et al., 2021). Table 1 presents the summary statistics for the TCR sequences.

### Simulation Strategy

We use a modified version of the simulation strategy as in (Degenhardt et al., 2019). The binary outcome is simulated based on a logistic regression model

$$\log\frac{Pr\left(Y=1\right)}{Pr\left(Y=0\right)}=\beta_{0}+\beta_{1}x_{1}+\beta_{2}x_{2}+\beta_{3}x_{3}$$

The three base variables ( $x_{1}$ , $x_{2}$ , and $x_{3}$ ) and three additional variables ( $x_{4}$ , $x_{5}$ , and $x_{6}$ ) are independently sampled from a uniform distribution of (0,1). The correlated predictor variables are simulated based on

$$v_{i}^{\left(j\right)}=x_{i}+(0.01+\frac{0.5(j-1)}{n_{i}-1})\times N(0,0.3)$$

for $j=1,\ldots ,g_{i}$ and i=1,…,6 , where $v_{i}^{\left(j\right)}$ denotes the j th variable in group i and $n_{i}$ is the size of group i . The correlation between the base variable $x_{i}$ and $v_{i}^{\left(j\right)}$ decreased as j increases. The additional predictor variables that are uncorrelated with any of those base variables and each other, $w_{k},\;\;k=1,\ldots ,\;\left(G-\overset{6}{\underset{i=1}{\sum }}g_{i}\right)$ , are also simulated based on a uniform distribution of (0,1), where G is the total number of the genes. Please note that $x_{i}$ , i=1,…,6, are only used to simulate correlated variables $v_{i}^{\left(j\right)}$ , and are not included for feature selection and classification. $v_{i}^{\left(j\right)}$ $j=1,\ldots ,g_{i}$ and i=1,2,3 , are the causal variables, while $v_{i}^{\left(j\right)}$ $j=1,\ldots ,g_{i}$ and $i=4,5,6\;\text{and}\;w_{k},\;\;k=1,\ldots ,\;\left(G-\overset{6}{\underset{i=1}{\sum }}g_{i}\right)$ are the non-causal variables.

We consider several different simulation scenarios (Table 2) including 1) different prevalence of the binary outcome ( η=0.25 and 0.5)  which is mainly determined by the coefficients in the logistic regression; 2) sparsity of causal genes (2.5%, 5%) with a different number of genes (G = 600 and 1,200); and 3) different sample sizes (*n* = 50 and 100). Under each scenario, the G genes consist of 30 causal ones $\left\{v_{i}^{\left(j\right)},i=1,2,3;\text{j}=1,\ldots ,10\right\}$ as well as 30 correlated, non-causal variables $\left\{v_{i}^{\left(j\right)},i=4,5,6;\text{j}=1,\ldots ,10\right\}\;$ and G−60 uncorrelated, non-causal variables $\left\{w_{k},k=1,\ldots ,G-60\right\}$ . For each of the scenarios,100 paired replicates are simulated. Each time the first one is used for feature selection and training the classifier, and the second is used for assessing stability and estimating prediction performance.

**TABLE 2:** Simulation scenario.

Label	Sample size	# of genes	P(Y = 1)	Sparsity (# of causal genes/# of genes)	$\beta_{1}$	$\beta_{2}$	$\beta_{3}$
n50_G600_eta0.5	50	600	0.5	30/600	−9	6	3
n50_G1200_eta0.5	50	1,200	0.5	30/1,200	−9	6	3
n100_G600_eta0.5	100	600	0.5	30/600	−9	6	3
n100_G1200_eta0.5	100	1,200	0.5	30/1,200	−9	6	3
n50_G600_eta0.25	50	600	0.25	30/600	−14	12	−6
n50_G1200_eta0.25	50	1,200	0.25	30/1,200	−14	12	−6
n100_G600_eta0.25	100	600	0.25	30/600	−14	12	−6
n100_G1200_eta0.25	100	1,200	0.25	30/1,200	−14	12	−6

### Existing Approaches of Feature Selection

Feature selection methods are often categorized into four classes: filters, wrappers, embedded, and hybrid methods. Filter methods evaluate and rank the importance of a single feature (univariate filter) or an entire subsect of features (multivariate filter) based only on their inherent characteristics, without incorporating any learning algorithm. Wrapper methods evaluate a specific subset of variables by training and testing a specific learning model (e.g., K-Nearest Neighbors (KNN) (Dudani, 1976) or Support Vector Machine (SVM) (Suykens and Vandewalle, 1999)). However, as the space of variables subset grows exponentially with the number of variables, the exhaustive search is very computationally intensive. Two alternative search schemes are commonly used to guide the search: sequential search, such as forward selection (add one at a time) or Recursive Feature Elimination (RFE, eliminate one at a time), and randomized search. Embedded methods consist of algorithms that simultaneously perform model fitting and feature selection. This approach is typically implemented using a sparsity regularizer or constraint on regression modeling, which shrinks the weight of some features to zero. Hybrid methods start with an initial feature filtering based on statistical properties, followed by a second selection based on wrapper methods. In this paper, we evaluate a variety of feature selection methods, including information gain (Kent, 1983) (univariate filter), correlation-based feature selection (Hall, 2000) (multivariate filter), SVM-RFE (Duan et al., 2005) (wrapper), Boruta (Kursa and Rudnicki, 2010) (wrapper), Vita (Malley et al., 2012) (wrapper), and LASSO (Tibshirani, 1996) (embedded). In addition, Boruta and Vita are built around Random Forest classifier and Random Forest is a bagging technique, therefore, Boruta and Vita can also be considered as feature selection approaches using bagging technique. The detailed information on those methods is provided in Supplementary Table S1. In addition to those listed above, there are a vast of feature selection methods existing in the literature. However, most of the time, each method selects different features, and it is difficult (almost impossible) to make a correct choice. Moreover, for small sample data (typical case for immune repertoire data), the selection based on a single feature selection method is usually not stable.

### A Novel Ensemble Feature Selection

**FIGURE 1:**
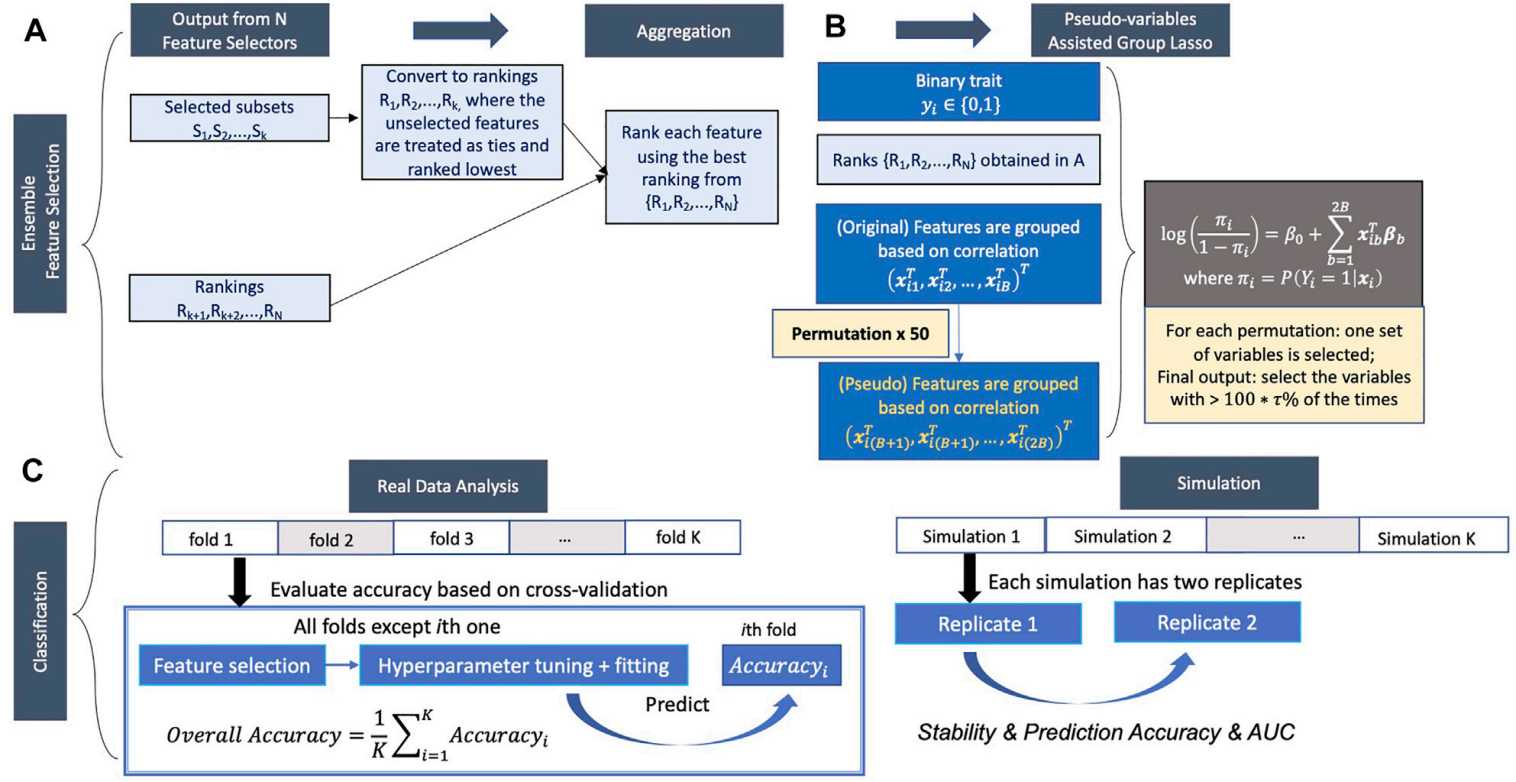
Proposed pipeline. **(A,B)** Ensemble feature selection. **(A)** Feature selection based on different methods and aggregation of selected features. **(B)** Pseudo-variables assisted group lasso. **(C)** Classification.

**FIGURE 2:**
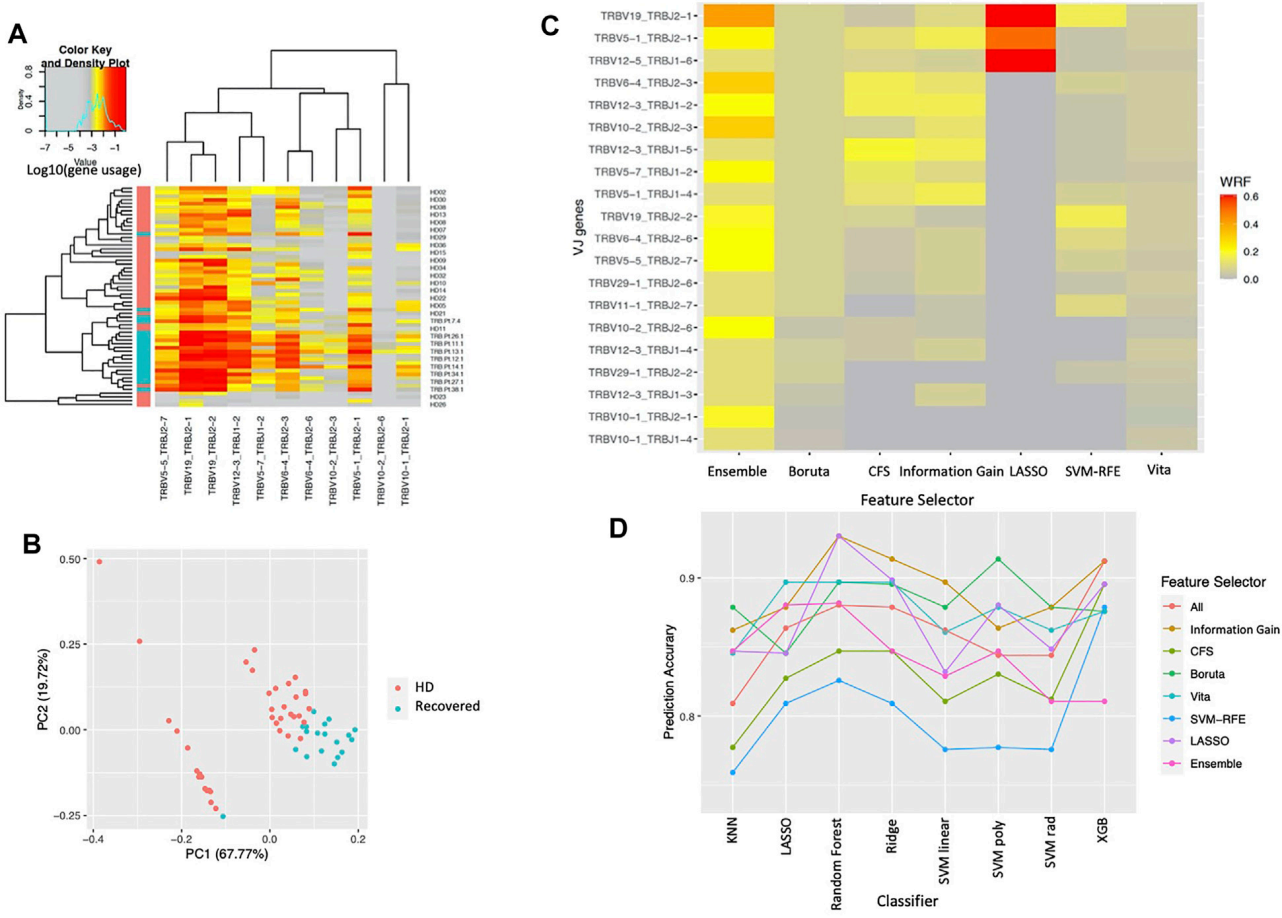
The results for COVID-19 dataset. **(A)** TCR VJ gene usage heatmap. VJ gene usage was assessed for each sample. Each column represents an individual single VJ gene combination, and each row represents an individual subject with red bar and green bar in left side of the heat map representing healthy donors (HD) and COVID-19 recovered patients (Recovered), respectively. The heat represents the VJ gene usage, with red to grey representing increased to decreased gene usage, respectively. **(B)** Principal components analysis based on the selected VJ genes. Each dot represents a single subject with red and green representing healthy donors (HD) and COVID-19 recovered patients (Recovered), respectively. **(C)** Normalized selection frequency of the top selected VJ genes by each feature selection approach. Each row represents an individual single VJ gene combination, and each column represents the feature selection approaches. **(D)** Prediction accuracy based on the selected VJ genes by each feature selection approach for different classification methods. Each line presents the prediction accuracy for the corresponding feature selection approach based on the corresponding classification method (x-axis).

Ensemble feature selection by combing the outputs from different feature selection methods can solve the problem mentioned above. Here, we propose a new ensemble feature selection procedure based on pseudo-variables, which has two major steps: 1) aggregating the feature selection results from multiple feature selectors (Figure 1A) and 2) fitting a group lasso model on the candidate feature set with a new permutation-assisted tuning strategy (Figure 1B). In the first step, we further expand to highly correlated features to generate a candidate set of features.

Aggregating the results from different feature selection approaches is a critical step in ensemble learning. The outputs of the different approaches can be various, either the subsets of selected features, the rankings of all features, or both. We consider the following general scheme to obtain the candidate feature set depending on the types of outputs (Figure 1A). Suppose the feature selection approach returns the subset output. In that case, the selected features will be first converted to ranking, where the selected features are treated as ties (unless there is order in output) and ranked highest (tied for the first place), and the unselected features are also treated as ties but rank lowest using the total number of features. The highest rank across the approaches (i.e., the best position that the feature achieved) is used to generate the aggregated ranking across all feature selection approaches for each feature. For example, if one feature ranks first and 10th in two approaches, first will be recorded as the aggregated ranking for this feature.

Now we introduce a group lasso model (Meier et al., 2008) on the candidate feature set (Figure 1B). Denote the total number of features included in the candidate feature set after aggregation and expansion is p . Assume observations after the aggregation and expansion are $\left(x_{i},y_{i}\right),i=1,\ldots ,n$ , where $x_{i}$ is of *p*-dimensional vector and $y_{i}\in \left\{0,1\right\}$ is a binary outcome. Without loss of generality, assume the *p* features are quantitative variables, but the method can be applied for categorical variables or a mixed type. By using the correlation structure of the *p* variables, we can define blocks 1,2,…,B such that within each block, the absolute value of pairwise correlation is all greater than a self-correlation threshold parameter $\rho_{T}$ . Assume *b*th block includes $L_{b}$ variables and $\overset{B}{\underset{b=1}{\sum }}L_{b}=p$ . The *p*-dimensional vector $x_{i}$ can be rewritten as $x_{i}={\left(x_{i1}^{T},x_{i2}^{T},\ldots ,x_{iB}^{T}\right)}^{T}$ with $x_{ib}$ of dimension $L_{b},\;b=1,\ldots ,\;B$ . We model the relationship between the binary $Y_{i}$ and features $x_{i}$ using the following logistic regression model

$$log\left(\frac{\pi_{i}}{1-\pi_{i}}\right)=\beta_{0}+\overset{B}{\underset{b=1}{\sum }}x_{ib}^{T}\beta_{b}≜\gamma_{\beta }\left(x_{i}\right),$$

with

$$\pi_{i}=P\left(Y_{i}=1\text{|}x_{i}\right),$$

where $\beta_{0}$ is the intercept, and $\beta_{b}\in R^{L_{b}}$ is the parameter vector for *b*th block. Denote the complete parameter vector by $\beta ={\left(\beta_{0},\beta_{1}^{T},\ldots ,\beta_{B}^{T}\right)}^{T}\in R^{p+1}.$ In the repertoire-sequencing data, our focus is to identify the crucial groups of VJ genes associated with the binary outcome, i.e., which $\beta_{b}\neq 0$ . The main challenge here is that the dimension of the VJ genes of repertoire data is usually very high (∼1,000) compared to the sample size (20–50). Despite the high dimensionality, we assume that only a small number of VJ gene groups impact the phenotype (i.e., a sparse model). Hence, the group-lasso-type methods (Meier et al., 2008) fit the scenario well because of their ability to shrink some of the coefficient vectors to precisely zero. The important VJ gene sets with notable effects on the phenotype will stand out. The estimation of the complete parameter vector β is given by minimizing the following objective function

$$Q_{\lambda }\left(\beta \right)=-l\left(\beta \right)+\lambda \overset{G}{\underset{b=1}{\sum }}s_{b}\|{\mathrm{\beta b}}_{\|2},$$

where l(β) is the log-likelihood function, i.e.,

$$l\left(\beta \right)=\overset{n}{\underset{i=1}{\sum }}y_{i}\gamma_{\beta }\left(x_{i}\right)-\log\left[1+\exp\left\{\gamma_{\beta }\left(x_{i}\right)\right\}\right],$$

And λ is the tuning parameter that controls the amount of shrinkage (larger lambda shrinks more to zero). The $s_{b}$ is used to rescale the penalty to each group and its default setting in group lasso methods was $\sqrt{L_{b}}$ . To put a small penalty on top-ranked feature sets, we propose using the product of the minimum rank among different feature selectors and $\sqrt{L_{b}}$ .

The selection of the tuning parameter λ of the group lasso model is typically performed by maximizing the cross-validation error (an estimate of prediction accuracy). However, the cross-validation error has a considerable variation when the sample size is small and potentially leads to less reliable conclusions (Varoquaux, 2018). The objective of this study is more about selecting the important features than improving the prediction accuracy. Therefore, we propose to use pseudo-variables (Candès et al., 2018) to facilitate the group-lasso tuning parameter selection. Let $X={\left(x_{1},x_{2},\ldots ,x_{n}\right)}^{T}=\left(X_{1},X_{2},\ldots ,X_{p}\right)$ be the original input features (VJ gene usage) matrix. The pseudo-variables matrix $X^{\pi }$ is generated through permutation, i.e., $X^{\pi }={\left(x_{\pi (1)},x_{\pi (2)},\ldots ,x_{\pi (n)}\right)}^{T}$ , where {π(1),π(2),…π(n)} is a permutation of {1,2,…,n}. Combining the original and permutated design matrixes, we define the augmented design matrix as $X^{A}=\left(X,X^{\pi }\right)$ of n rows and 2 p columns, where the first p columns are original input features and second p columns are pseudo features (but preserve the correlation of the structure of original features). The optimization problem corresponding to group lasso fitting after augmentation becomes

$$\beta^{A}\left(\lambda \right)=\text{a}\text{r}\text{g}\text{m}\text{i}{\text{n}}_{\beta^{A}}\;\;-l\left(\beta^{A}\right)+\lambda \overset{2B}{\underset{b=1}{\sum }}s_{b}\|\beta_{\mathrm{A,b}\|2}^{T}$$

where $\beta^{A}=$ ${\left(\beta_{A,0},\beta_{A,\;1}^{T},\ldots ,\beta_{A,\;B}^{T},\beta_{A,\;B+1}^{T},\ldots ,\beta_{A,\;2B}^{T}\right)}^{T}\in R^{2p+1}$ is the regression coefficients vector including the intercept, B sets of original VJ genes and B sets of pseudo-VJ genes. Given a tuning parameter λ , the estimated $\hat{\beta^{A}}\left(\lambda \right)$ can be obtained by solving a convex optimization problem. As λ increases, more blocks of coefficient vectors $\beta_{A,b}\;$ shrink to zero (i.e., fewer groups remain in the model), and the most important group shrinks lastly. For each (either original or pseudo) set, define $R_{b}=\sup\left\{\lambda :\beta_{A,\;b}^{T}\neq 0\;\right\},\;b=1,\ldots ,\;2B,$ which can be viewed as an importance measure of the feature set. The larger $R_{j}$ is, the more important the set is. Then define $T_{\pi }=\underset{\left(\text{B}+1\right)\leq \text{b}\leq 2\text{B}}{\max}R_{b}$ , which can be utilized to separate the active features from the inactive artificial ones. Based on the value “benchmark” $T_{\pi }$ , the selection for each permutation can be made with ${\hat{S}}_{\pi }=\left\{b:R_{b}>T_{\pi },\;b=1,\ldots ,B\right\}$ , i.e., selecting the original sets which are more important than the artificial ones. Repeat this process for *K* times and report the feature sets selected more than a certain percentage threshold τ (e.g. 50%).

### Integrated Feature Selection and Classification Pipeline

We then feed the selected features (based on six existing methods and the novel ensemble feature selection approach) into eight different classifiers, including SVM with linear (SVM linear), polynomial (SVM poly) and radius kernels (SVM rad) (Amari and Wu, 1999), K-nearest neighbors (KNN) (Dudani, 1976), Random Forest (Breiman, 2001), extreme gradient boosting (XGB) (Chen et al., 2015), ridge (Le Cessie and Van Houwelingen, 1992) and LASSO (Tibshirani, 1996).

### Performance Evaluation

For simulation studies, we assess and compare the performance of the different variable selection approaches by using the following measures: false discovery rate (FDR), sensitivity, stability, F-1 score (Hua et al., 2009), and empirical power (Figure 1C). For each method, within each replicate, FDR is calculated as the ratio of the number of false-positive results, i.e., the total number of non-causal variables ( $v_{i}^{\left(j\right)}$ $j=1,\ldots ,g_{i}$ and $i=4,5,6\;\text{and}\;w_{k},\;\;k=1,\ldots ,\;\left(G-\overset{6}{\underset{i=1}{\sum }}g_{i}\right)$ ) selected to the total number of variables selected. In contrast, sensitivity is defined as the proportion of correctly identified causal variables ( $v_{i}^{\left(j\right)}$ $j=1,\ldots ,g_{i}$ and i=1,2,3 ) among all causal variables per replicate and method. F-1 score is calculated as 2*(precision*sensitivity)/(precision + sensitivity), a balance (the harmonic mean) of precision and sensitivity, where precision = 1-FDR (Hua et al., 2009). For each pair of replicates, the Jaccard’s index is calculated as the ratio of the length of the intersection and the length of the union of the two sets of selected variables (He and Yu, 2010). The average across all pairs is used to quantify the stability of variable selection for the particular method (Kalousis et al., 2007). The empirical power of each causal variable ( $v_{i}^{\left(j\right)}$ $j=1,\ldots ,g_{i}$ and i=1,2,3 ) is calculated as the frequency of correct selections among all replicates (Dash and Liu, 1997). The prediction accuracy and area under the curve (AUC) are assessed on the paired replicate to evaluate the performance of the classification (Huang and Ling, 2005). The parameters we used were listed in Table 3.

**TABLE 3:** Parameters used for feature selection methods.

Approach	R Package	Parameter	Description	Value
Information gain	FSelector	k	Select top k features	0.05 × G
CFS	FSelector	default		
Boruta	Boruta	Final Decision	Three possible values as the final decision	Confirmed /tentative
Vita	Vita	k	Cross-validation fold	5
		pvalue threshold	Selection criteria of pvalue	0
SVM_RFE	mSVM-RFE	k	Cross-validation fold	5
		selection criteria	Top features	0.05 × G
LASSO	glmnet	lamba	Tunning parameter grid values	10^(−10,−9.9,…,0,…,9.9,10)^
Ensemble		$\rho_{T}$	Minimum pairwise correlation within block	0.75
		K	Total number of permutations	50
		τ	Threshold of selection percentage	0.5

G is the total number of the features.

In the real data analysis, we use 5-fold cross-validation to evaluate the prediction accuracy and AUC (Figure 1C). Feature selection, model fitting, and parameter tuning are performed using the four folds of the data, and prediction accuracy and AUC are evaluated by averaging the values on the held-out fold data. However, because the causal variables in real data are unknown, we can’t assess FDR, sensitivity, F-1 score, and empirical power of the feature selection approaches. The relationship of features selected in different feature selection methods was investigated, and the most frequently selected features in each fold among all methods for both datasets were also evaluated. Considering each feature selection output varies in the number of features selected, we used a weighted relative frequency (WRF) to measure the relative frequency that a feature is selected across five different folds. Specifically, $WRF_{j}=\overset{5}{\underset{f=1}{\sum }}I\left\{X_{j}\in S_{f}\right\}/n_{f}$ for j th feature, where $S_{f}$ is the set of selected features using all but f th fold data and $n_{f}$ is the total number in $S_{f}$ . $I\left\{X_{j}\in S_{f}\right\}$ is an indicator function which takes value 1 if feature $X_{j}$ is one of the selected features and takes value 0 if not. For example, if the five selections are { $X_{1},\;X_{2}$ }, { $X_{2},\;X_{4},\;X_{5}$ }, { $X_{1},\;X_{5}$ }, { $X_{1}$ } and { $X_{2},\;X_{5},\;X_{7}$ }, then $WRF_{1}$ = 1/2 + 0/3 + 1/2 + 1/1 + 0/3 = 2.

All the analyses were performed by R (https://www.r-project.org).

## Results

### Ensemble Feature Selection Approach Efficiently Selected Key Features on Real Data Analysis

We classified clonotypes into 708 VJ gene combinations and assessed whether VJ gene usage within the T-cell repertoire differed between the two cohorts in the COVID-19 dataset. Our proposed ensemble method shows the gene usage of 11 VJ genes that were all significantly higher in the COVID-19 recovered patients compared to their usage in healthy donors (Figure 2A). The 11 genes are selected at least twice in the 5-folds cross-validation procedure (Table 1), including a more significant increase in the TCRV5-1/J2-1, V5-5/J2-7, V6-4/J2-3, V12-3/J1-2, V19/J2-1, and V19/J2-2 gene usages in COVID-19 recovered patients, which were reported in the original paper (Schultheiß et al., 2020). The principal components analysis (PCA) based on the 11 selected VJ genes shows that the two cohorts can be segregated mainly by these 11 VJ usages (Figure 2B). In addition, we compared the selection frequency across the different feature selection approaches by cross-validation. We did 5-fold cross-validation within each approach and calculated the weighted relative frequency for each gene. Figure 2C shows the heatmap of the top 20 selected genes ordered by their WRFs based on the ensemble method, for different feature selection methods. It can be observed that lasso can only identify the top signals with strong signals (hence those variables have large WRFs), and the proposed feature ensemble method can aggregate the top signals identified by the existing approaches. Figure 2D presents the prediction accuracy of the selected genes based on the different feature selection methods for different classifiers. We found that all feature selection approaches (including the feature ensemble method) have very similar prediction accuracy in terms of classification performance comparing to the results without feature selection.

In addition, we considered to identify the VJ genes in the lung cancer dataset based on the patients’ overall survival time. There are 17 longer survivors and 33 short survivors, which were defined based on longer or shorter than the median overall survival (20.3 months), respectively. Because the lung cancer patients received durvalumab, we selected the VJ genes based on their usage changes from baseline to the post-treatment, which was defined as the ratio of VJ gene usages from post-treatment vs. the usages from baseline. We identified 9 genes: TRBV5-3/J1-1, TRBV1/J2-7, TRBV1/J1-5, TRBV20-1/J1-4, TRBV7-4/J2-3, TRBV11-1/J2-6, TRBV7-7/J2-2, TRBV1/J1-1, and TRBV5-7/J1-6 when long survivors compared to short survivors (Supplementary Figure S1).

### Ensemble Feature Selection Approach Consistently Outperformed on Simulation Studies

**FIGURE 3:**
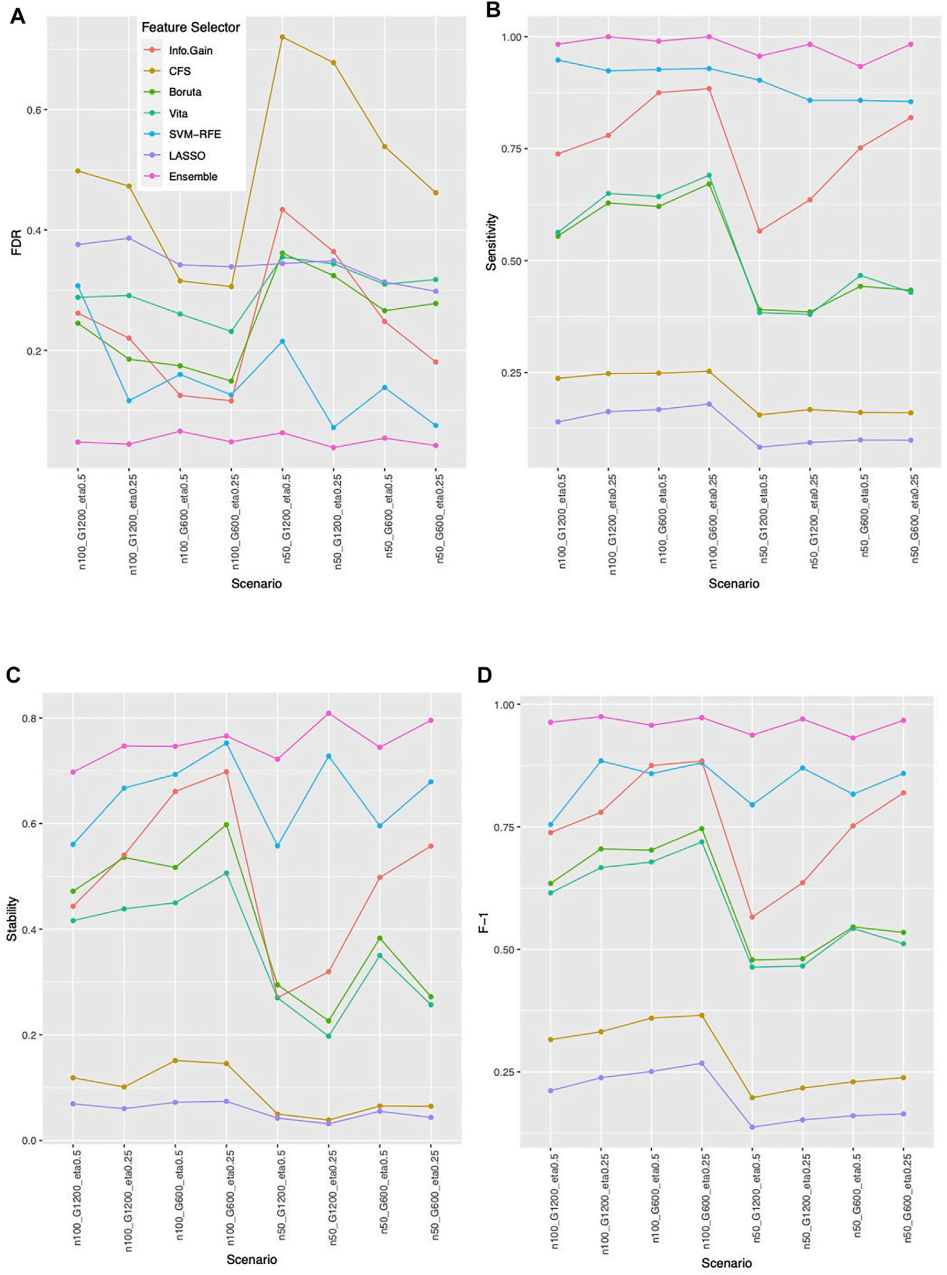
Feature selection performance based on simulation. **(A)** FDR **(B)** Sensitivity **(C)** Stability **(D)** F-1. In each panel, x-axis stands for different simulation scenario listed in Table 2. For example, n50_G600_eta0.5 stands for sample size is 50 with 600 candidate genes and the probability of the outcome is 0.5. Each colored curve stands for different feature selection approaches.

**FIGURE 4:**
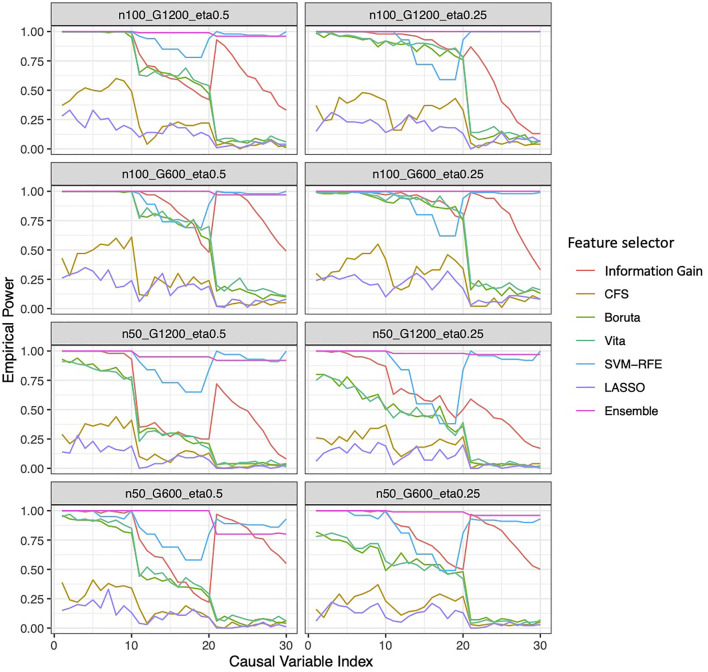
Empirical power of the feature selection approaches based on simulation. Each panel represents different simulation scenario listed in Table 2. For example, n50_G600_eta0.5 stands for sample size is 50 with 600 candidate genes and the probability of the outcome is 0.5. In each panel, x-axis stands for the number of causal variables.

In general, the ensemble feature selection approach consistently outperforms the other state-of-the-art feature selection methods in terms of both stability and accuracy. It possesses consistent higher stability and sensitivity but lower FDR independent of the sample size choices, the sparsity of the causal genes, and the prevalence of the outcomes (Figure 3). As expected, a larger sample size increases the stability, sensitivity and F-1 score, but almost didn’t change FDR. Interestingly, a higher outcome prevalence results in lower stability for most methods except lasso and CFS, higher FDR and lower sensitivity. More genes in the pool introduce less stability, slightly more FDR and almost no change in sensitivity. Together with LASSO, the ensemble method is relatively robust to the choices of sample size, the sparsity of the causal genes, and the prevalence of the outcomes in terms of F-1 score, sensitivity and FDR. However, the performance of LASSO is always much worse than the ensemble method. In addition, the proposed ensemble approach always maintains the largest power (close to 1) in all simulation scenarios while some approaches could have as low as less than 50% of power (Figure 4). And the power that the ensemble approach can achieve is robust to the number of causal variables in the simulation, unlike the traditional approaches, the power is significantly impacted by the number of the causal variables.

**FIGURE 5:**
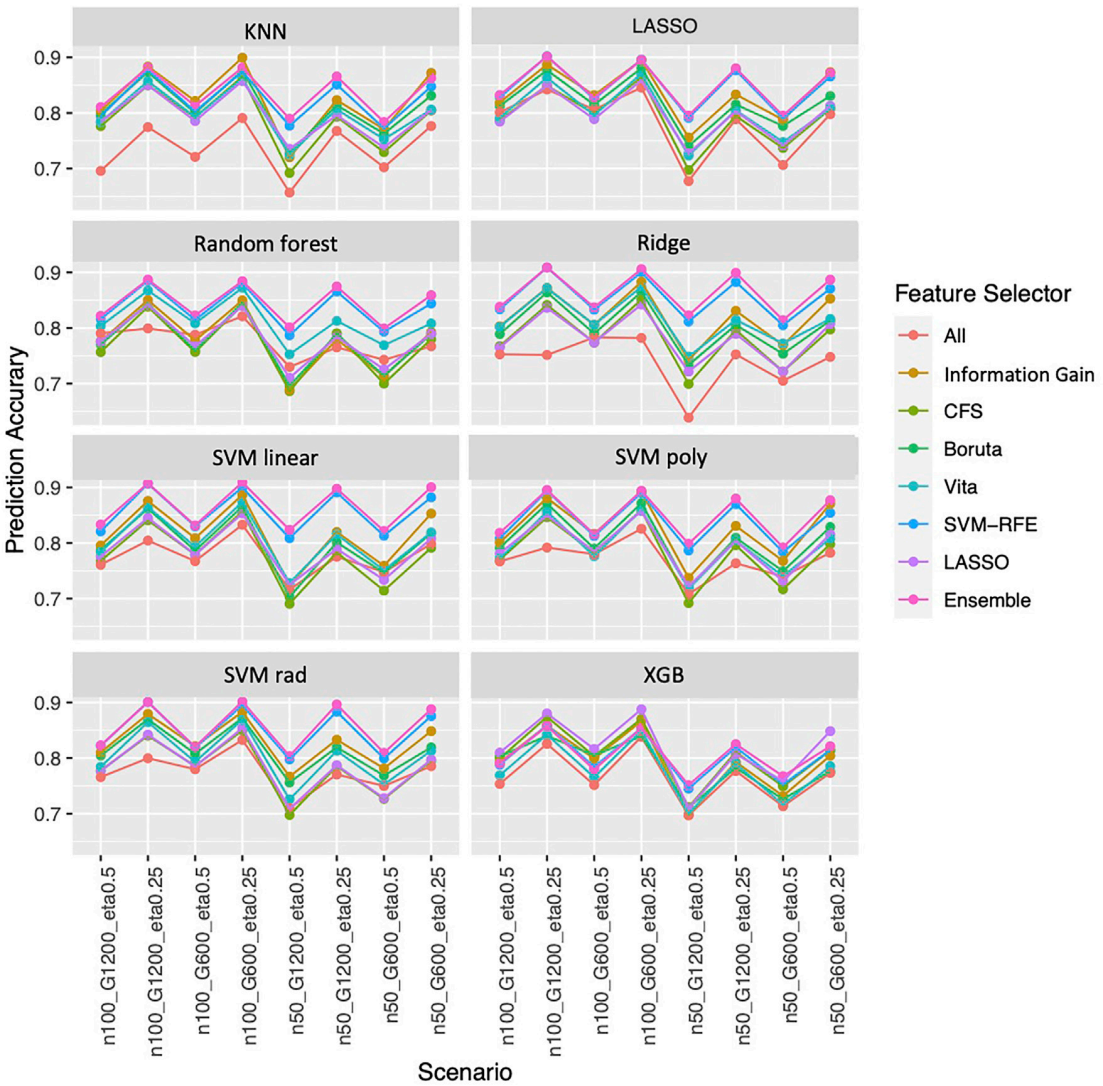
Prediction accuracy based on simulation. Panels present the prediction accuracy for the corresponding classification approach as indicated. In each panel, x-axis stands for different simulation scenario listed in Table 2. For example, n50_G600_eta0.5 stands for sample size is 50 with 600 candidate genes and the probability of the outcome is 0.5. Each colored curve stands for different feature selection approaches. The eight classification approaches are: SVM with linear (SVM linear), polynomial (SVM poly) and radius kernels (SVM rad) (Amari and Wu, 1999), K-nearest neighbors (KNN) (Dudani, 1976), Random Forest (Breiman, 2001), extreme gradient boosting (XGB) (Chen et al., 2015), Ridge (Le Cessie, Van Houwelingen) and LASSO (Tibshirani, 1996).

Overall, the ensemble feature selection also improves the classification performance. The ensemble feature selection approach has the best prediction accuracy when integrating with LASSO, Random Forest, ridge, and SVM classification methods. While combing with KNN, the ensemble feature selection approach occasionally is not as good as information gain performs, but most of the time is worse. When working with xgb, the ensemble feature selection approach has competitive performance compared to LASSO (Figure 5). The ensemble feature selection approach has the highest AUC except when interpreting with KNN and Random Forest, and it has competitive performance compared to information gain (Supplementary Figure S2). Similarly, a larger sample size or a smaller number of genes increases the prediction accuracy while a higher outcome prevalence results in lower prediction accuracy (Figure 5). However, the influence on AUC is relatively small for the ensemble method (Supplementary Figure S2).

## Discussion

We formulated a novel ensemble feature selection approach with a customized statistical learning algorithm focused on VJ gene usage in repertoire-sequencing data. Using the proposed approach and algorithm, we identify the VJ genes with significantly different usage in COVID-19 recovered patients and healthy donors. Wang et al. analyzed the TCR repertoire in patients with COVID-19 using single-cell sequencing and found that the frequencies of TRAV4, TRAJ2-7, TRBV7-9, and TRBJ2-3 were significantly higher compared to healthy patients (Wang et al., 2021). We found that the TCR beta chains TCRV5-1/J2-1, V5-5/J2-7, V6-4/J2-3, V12-3/J1-2, V19/J2-1, and V19/J2-2 had higher frequencies among patients with COVID-19. Overall, identifying these VJ genes could reflect a specific antigen milieu leading to the selection of a distinct combination of VJ genes. Further correlation of these unique VJ gene profiles with clinical outcomes can potentially aid in the development of sorely needed prognostic tools for patients infected with COVID-19. Additionally, among the 9 VJ gene usages identified in lung cancer patients treated with Durvalumab, one of the identified V segments, TRBV20-1 has been previously shown to be differentially expressed in cancer tissue compared to healthy tissue (Wang et al., 2019). Furthermore, TRBV20-1 usage has been associated with improved response and survival in lung cancer patients treated with anti-PD1 therapy such as Durvalumab (Dong et al., 2021). Thus, the other identified VJ gene segment pairs above should be explored as potential additional features of the TCR repertoire associated with improved clinical response.

In the real data analysis, we found that none of the feature selection approaches (including the feature ensemble method) have substantial improvements in terms of classification performance comparing to the results without feature selection. This is not surprising, since feature selection is not guaranteed to improve the prediction accuracy. However, feature selection is able to reduce the dimensinsionality and complexity of the predictive models, which eventually leads to a faster model trainning time and convergency. Our ensemble method, though is not driven by the prediction accuracy as some other feature selectors (e.g., SVM-RFE, Boruta, Vita) is still able to exhibit a competitive performance in spite of a small sample size and even with highly correlated features included.

In addition, we carry out intensive simulation studies in different scenarios. We found that the ensemble feature selection approach surpasses the other commonly used feature selection methods based on efficiency and accuracy. When integrating with varying types of classification methods, in most cases, the ensemble feature selection approach has the best prediction performance. These results indicate that the ensemble feature selection approach not only identifies the most stable, highest sensitive features with low false discovery rates but also greatly improves the prediction performance. Sample size, sparsity of causal genes, and the prevalence of the outcomes influence the performance but are relatively small for the ensemble approach.

In the similation studies shown above, the base learners used in the first phase were information gain, SVM-RFE, Vita and Boruta. We have conducted additional simulation studies, where the different base learners were used. The results shown that our proposed ensemble method is neither sensitive to the number nor to the choice of base learners (Supplementary Table S2). In addition, we found that the proposed method is also relatively robust to the choices of those parameters based on the simulation studies (Supplementary Table S3). Note that the large threshold $\rho_{T}$ will introduce singleton groups and small threshold will introduce large groups, which may impact the variable selection results. Very large groups, unless very strong signal, will less likely to be included in the selection, because the proposed group lasso model penalizes based on the group size. In an extreme case of all groups are singleton, it is reduced to a regular lasso model, where only one variable might got picked among highly correlated variables. Therefore, a moderate threshold is recommended. In our case, we set the correlation threshold of 0.75 to keep the highly correlated variables in the same block while the uncorrelated variables in singleton groups.

Though the repertoire-sequencing data was used to illustrate this approach, the proposed approach can be applied to any other feature selection work. In a real application, we could perform stratified feature selection within the strata defined by the important covariates or consider the covariates as additional features. Although the ensemble feature selection was currently applied to a binary outcome, it can also be extended to different types of outcomes (continuous, multi-level categorical outcomes, and time-to-event outcomes) by changing the log-likelihood in the objective function in optimization. Not surprisingly, the proposed ensemble method, which aggregates the output from multiple feature selectors, takes longer than a single feature selection method. However, it has been demonstrated in the simulation studies that the feature selection performance can be significantly boosted. Moreover, we want to point out that the proposed method can be applied as an independent feature selection method by setting the rank matrix with all elements equal to a constant. In addition, by using parallel computing, the computational time can be dramatically decreased. Furthermore, learning in a small n large *p* case is always challenging due to the minimal information observed in the high dimensional space. However, our simulation shows that the performance of both feature selection and classification is still appealing.

In conclusion, the proposed novel approach and integrated procedure can help us pursue an effective feature selection technique to aid in correctly prioritizing the important features and classifying different subtypes.

## Data Availability

R codes are available on GitHub (https://github.com/mlizhangx/Ensemble-Feature-Selection). The COVID data is available via gateway.ireceptor.org; Study ID: IR-Binder-000001. The lung cancer data underlying the findings described in this article may be obtained in accordance with AstraZeneca’s data sharing policy described at https://astrazenecagrouptrials.pharmacm.com/ST/Submission/Disclosure.
