# Supplementary material for: Novel Ensemble Feature Selection Approach and Application in Repertoire Sequencing Data
Source: Front Genet. 2022 Apr 26;13:821832. doi: 10.3389/fgene.2022.821832 (PMC9086194; doi:10.3389/fgene.2022.821832)
Supplement: Supplementary file 1 [file Table1.docx]

Table S1: Summary of the different variable selection methods

| Method | Category | Tasks | Output | Approach | R package |
| --- | --- | --- | --- | --- | --- |
| Correlation-based Feature Selection (CFS) | Multivariate filter method | Classification, Regression | A subset of variables (based on an appropriate correlation measure) | This method samples randomly instances from the dataset and updates the relevance of each feature based on the difference between the selected instance and the two nearest instances of the same and opposite classes. If a feature difference is observed in the neighboring instances of the same class (a ‘hit’), the feature score decreases, alternatively if the feature value difference is observed with a different score (a ‘miss’) then the feature score increases. There are variations of CFS that employ different attribute quality measures, such as Symmetrical Uncertainty, normalized symmetrical Minimum Description Length (MDL), or Relief [15,16]. | cfs() function in R package {FSelector} [1,2]: The algorithm finds attribute subset using correlation and entropy measures for continuous and discrete data. |
| Information Gain | Univariate filter method | Classification | A rank of variables (based on entropy) | Algorithms that find ranks of importance of discrete attributes, basing on their entropy with a class attribute | Information.gain() function in R package {FSelector} [1,2]: the algorithm finds weights of discrete attributes basing on their correlation with continuous class attribute. |
| Support Vector Machine Recursive Feature Elimination (SVM-RFE) | Wrapper method | Classification | A rank of variables (based on model evaluation metrics such as accuracy and MSE) | Combines both recursive feature elimination and support vector machine model construction. Recursive feature elimination works by repeatedly constructing models with all, but one variable and measuring the predictive accuracy of those models to determine a weight for each predictor. The feature with the lowest weight is then removed and the process is repeated to gain a ranking of each predictor significance. The final output of this algorithm is a ranked list with variables ordered according to their relevance. | R package {caret}[3]: rfe() function in R package {caret}: a simple backward selection, a.k.a recursive feature elimination (RFE), algorithm. |
| Boruta | Wrapper method | Classification, Regression | A subset of variables (based on comparison of Z-scores between real variable and shadow variables) | Compare the importance of the real variables to the shadow variables that are obtained by permuting a copy of the real variables across observations to destroy the relationship with the outcome. Subsequently a RF is trained on the extended data set, the permutation importance values are collected and for each real variable the respective importance is compared to the maximum value of all shadow variables using a statistical test. The unimportant variables are removed and the whole process is replicated until all variables are labeled or a predefined number of runs has been performed. | Boruta() function in R package {Boruta} [4]: the method performs a top-down search for relevant features by comparing original attributes’ importance with importance achievable at random, estimated using their permuted copies, and progressively eliminating irrelevant features to stabilize that test. |
| Vita | Wrapper method | All relevant variables | A rank of variables (ordered by p-values) | P-values based on empirical null distribution based on non-positive importance scores calculated using hold- out approach.  Most variables in omics data sets are unimportant and can be used to estimate importance values of null variables. | CVPVI() function in R package {vita} [5]: computes cross-validated permutation variable importance measure from a random forest or classification and regression.  NTA() function in R package {vita} [5]: calculates the p-values for each permutation variable importance measure, based on the empirical null distribution from non-positive importance values. |
| Least Absolute Shrinkage and Selection Operator  (LASSO) | Embedded method | Classification, Regression | A subset of variables (based on the results of penalized regression) | Lasso regression uses an L1 penalty, which not only shrinks coefficients towards zero, but also forces some of them to be exactly zero, which completely eliminates this variable altogether. A smaller subset of features that optimize for accuracy rates while reducing variance and increasing interpretability. | glmnet() function in R package {glmnet} [6]: Fit a generalized linear model via penalized maximum likelihood.  cv.glmnet() function in R package {glmnet} [6]: does k-fold cross-validation from glmnet. |

Table S2: Performance comparison between different feature ensembles under scenario: sample size of 50 with 600 candidate genes and the probability of the outcome is 0.5.

| Different ensembles | Compared to the ensemble used in the simulation:  Info.Gain + Boruta + Vita + SVM-RFE | | | | | |
| --- | --- | --- | --- | --- | --- | --- |
|  | $\Delta$FDR | $\Delta$Sensitivity | $\Delta$F-1 | $\Delta$Stability | $\Delta$Power^*^ | $\Delta$Accuracy^**^ |
| Info.Gain + SVM-RFE | +0.011 | -0.048 | -0.045 | -0.051 | -0.05  [-0.1,0] | +0.0025  [-0.004,0.005] |
| Info.Gain + Boruta | 0 | +0.017 | +0.009 | -0.008 | 0  [-0.035,0.1] | +0.001  [-0.003,0.011] |
| Info.Gain + SVM-RFE + Vita | +0.0069 | -0.015 | -0.015 | -0.031 | 0  [-0.045,0] | +0.003  [-0.001,0.017] |
| Info.Gain + Boruta + Vita | 0 | -0.017 | -0.016 | -0.0075 | 0  [-0.04,0] | +0.0005  [-0.003,0.011] |
| Info.Gain + Boruta + Vita + SVM-RFE + LASSO | -0.0017 | +0.02 | +0.014 | +0.011 | 0  [0,0.049] | +0.001  [-0.006,0.009] |

^*^Median and range of power change over 30 causal variables are calculated

^**^ Median and range of accuracy change over 8 different classifiers are calculated

Table S3: Performance comparison by varying different parameter of feature ensemble under scenario: sample size of 50 with 600 candidate genes and the probability of the outcome is 0.5.

| Parameter values | Compared to the default setting: $\tau=0.5, K=50, \rho_{T}=0.75$ | | | | | |
| --- | --- | --- | --- | --- | --- | --- |
|  | $\Delta$FDR | $\Delta$Sensitivity | $\Delta$F-1 | $\Delta$Stability | $\Delta$Power^*^ | $\Delta$Accuracy^**^ |
| $\tau=0.4$ | 0 | 0 | 0 | 0 | 0 [0,0] | 0.0027 [-0.02,0.011] |
| $\tau=0.6$ | 0 | 0 | 0 | 0 | 0 [0,0] | 0 [-0.015,0.018] |
| $K=100$ | 0 | 0 | 0 | 0 | 0 [0,0] | 0 [-0.011,0.021] |
| $K=200$ | 0 | 0 | 0 | 0 | 0 [0,0] | -0.0013 [-0.031,0.013] |
| $\rho_{T}=0.65$ | -0.001 | +0.033 | +0.022 | +0.005 | 0 [0,0] | +0.0027 [-0.02,0.019] |
| $\rho_{T}=0.85$ | +0.001 | -0.0102 | -0.009 | -0.019 | 0 [0,0] | +0.003 [-0.009,0.022] |

^*^Median and range of power change over 30 causal variables are calculated

^**^ Median and range of accuracy change over 8 different classifiers are calculated

**Reference**

1. Yu and Liu, 2003: Feature Selection for High-Dimensional Data: A Fast Correlation-Based Filter Solution.
2. Y. Wang, I.V. Tetko, M.A. Hall, E. Frank, A. Facius, K.F.X. Mayer, and H.W. Mewes, "Gene Selection from Microarray Data for Cancer Classification<U+2014>A Machine Learning Approach," Computational Biology and Chemistry, vol. 29, no. 1, pp. 37-46, 2005.
3. Duan KB, Rajapakse JC, Wang H, Azuaje F. Multiple SVM-RFE for gene selection in cancer classification with expression data. IEEE Trans Nanobioscience. 2005 Sep;4(3):228-34. doi: 10.1109/tnb.2005.853657. PMID: 16220686.
4. Kursa M, Rudnicki W. Feature selection with the Boruta pack- age. J Stat Softw 2010;36:1–13.
5. Janitza, Silke & Celik, Ender & Boulesteix, Anne-Laure. (2016). A computationally fast variable importance test for random forests for high-dimensional data. Advances in Data Analysis and Classification. 12. 10.1007/s11634-016-0276-4.
6. Friedman J, Hastie T, Tibshirani R (2010). “Regularization Paths for Generalized Linear Models via Coordinate Descent.” Journal of Statistical Software, 33(1), 1-22.
