# Supplementary material for: Novel Ensemble Feature Selection Approach and Application in Repertoire Sequencing Data
Source: Front Genet. 2022 Apr 26;13:821832. doi: 10.3389/fgene.2022.821832 (PMC9086194; doi:10.3389/fgene.2022.821832)

Supplementary Figure 2. Area under the receiver operating characteristic curve (AUC) based on simulation. Panels present the AUC for the corresponding classification approach as indicated. In each panel, x-axis stands for different simulation scenario listed in Table 2. For example, n50_G600_eta0.5 stands for sample size is 50 with 600 candidate genes and the probability of the outcome is 0.5. Each colored curve stands for different feature selection approaches. The eight classification approaches are: SVM with linear (SVM linear), polynomial (SVM poly) and radius kernels (SVM rad) [19], K-nearest neighbors (KNN) [9], Random Forest [20], extreme gradient boosting (XGB) [21], Ridge [22] and LASSO [16].

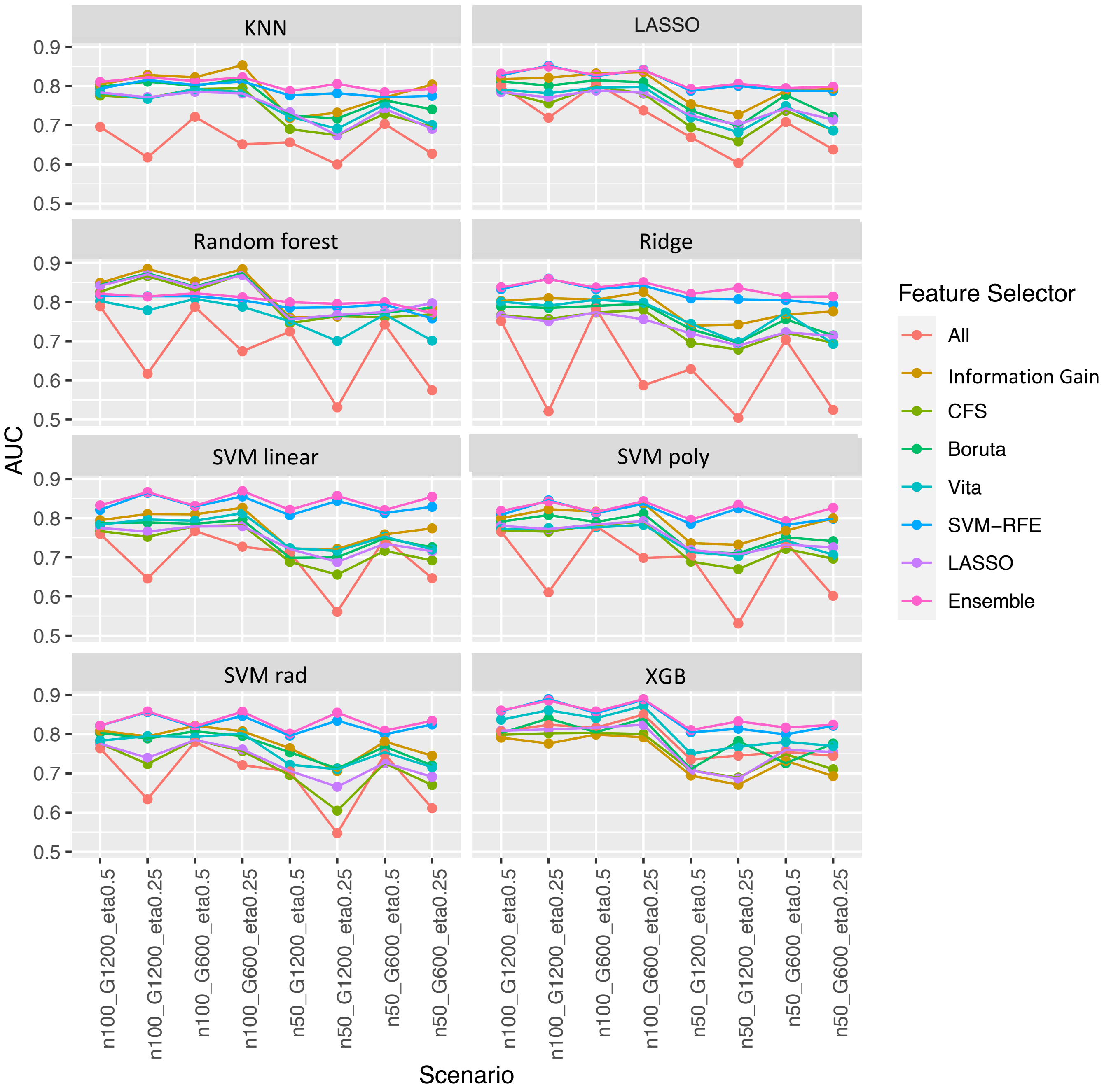

Supplement: Supplementary file 2 [file Image2.pdf]
