# Supplementary material for: Novel Ensemble Feature Selection Approach and Application in Repertoire Sequencing Data
Source: Front Genet. 2022 Apr 26;13:821832. doi: 10.3389/fgene.2022.821832 (PMC9086194; doi:10.3389/fgene.2022.821832)

Supplementary Figure 1. The results for lung cancer dataset. A) TCR VJ gene usage heatmap. VJ gene usage was assessed for each sample. Each column represents an individual single VJ gene combination, and each row represents an individual subject with red bar and green bar in left side of the heat map representing longer survivors (Long) and longer survivors (Short), respectively. The heat represents the VJ gene usage, with red to grey representing increased to decreased gene usage, respectively. B) Principal components analysis based on the selected VJ genes. Each dot represents a single subject with red and green representing longer survivors (Long) and longer survivors (Short), respectively. C) Selection frequency of the top selected VJ genes by each feature selection approach. Each row represents an individual single VJ gene combination, and each column represents the feature selection approaches.

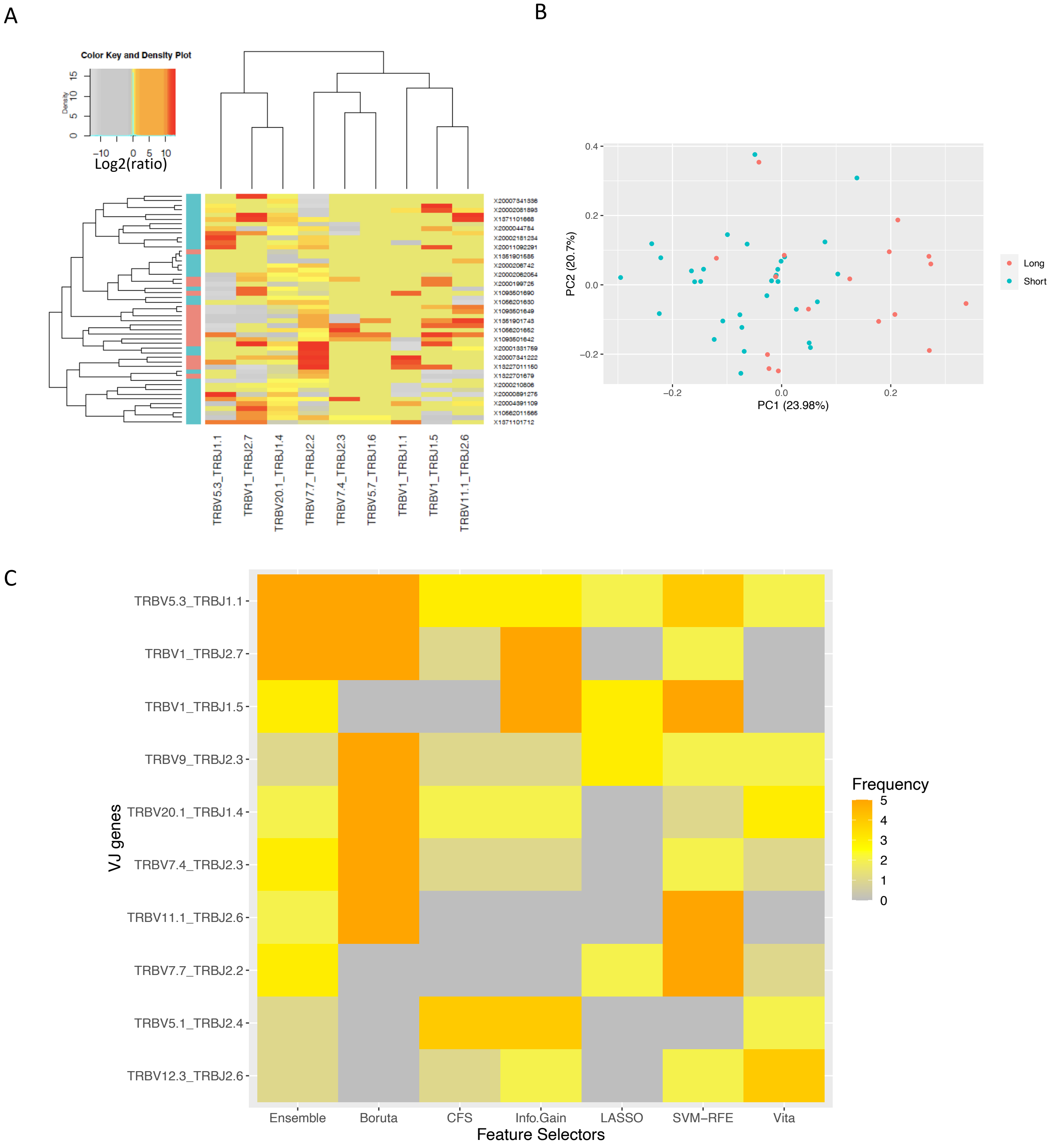

Supplement: Supplementary file 3 [file Image1.pdf]
